# Validating LA/AIDS model in the food market of Pakistan

**DOI:** 10.1016/j.heliyon.2022.e10699

**Published:** 2022-09-19

**Authors:** Ghulam Mustafa, Weidong Huo, Amber Pervaiz, Muhammad Rizwan Ullah, Muhammad Zulfiqar

**Affiliations:** a Department of Economics, University of Education, Lahore, Pakistan; b School of Finance and Trade, Liaoning University, Shenyang, China; c Lyallpur Business School, Government College University, Faisalabad, Pakistan

**Keywords:** Linear approximate almost ideal demand system, Food demand, Own price elasticities, Cross-price elasticities, Expenditure elasticities, Income elasticities, Pakistan

## Abstract

The study aims to conduct a consumer demand analysis of the food market of Pakistan by estimating its own price and cross-price elasticities. This study also examines expenditure and income elasticities to show the influence of relative change in price, total expenditure, and income on the relative change in demanded quantities of the selected food products. The study takes meat, vegetables, fruits, and pulses as different food baskets and estimates income elasticities, including uncompensated (Marshallian) and compensated (Hicksian) own price and cross-price elasticities. The findings are concluded based on Marshallian elasticity as it provides more accurate images of substitutes and complements compared with Hicksian elasticity. The study applies the Linear Approximate Almost Ideal Demand System model to estimate the results by acquiring data from a household integrated economic survey of Pakistan from 2018 to 2019. The findings of expenditure elasticity (uncompensated own price elasticity) reveal that vegetables and pulses are normal (inelastic) goods, whereas meat and fruits are luxury (elastic) goods. The results of uncompensated cross-price elasticities reveal that vegetables and meat, and vegetables and fruits are substitutable commodities. In addition, pulses and vegetables, and pulses and meat are complementary goods. The study suggests fruitful implications for food policymakers.

## 1 Introduction

Consumer demand analysis has been a topic of pivotal importance for many years. Over the past few decades, this field has received sustained research activity by different academicians, even at present. Consumer demand analysis is a very active and bourgeoning research area in developed and developing economies. Recently, a rapid increase in food prices attracted the attention of economic researchers to empirically analyze the behavior of consumers (Mudassar et al., 2012). The literature documented unrelenting research efforts of different economists to conduct their studies on the specific demand elasticity estimates. Incontestably, income and price elasticities not only provide an understanding of consumer behaviors but also broaden the vision for the policy analysis (Mudassar et al., 2012).

The question of how household adjusts their consumption patterns in response to changes in income and price encourages several researchers to conduct studies in different contexts to observe the behavioral pattern of consumers. Different researchers, academicians, and policymakers strongly believe that the development of an economy is highly correlated with the consumption pattern of households as consumption patterns make welfare analysis easier (Ullah, 2018; Akram, 2020). The consumption patterns of households are also useful in business progress as the whole investment setup relies on the consumption patterns of a country (Akram, 2020).

According to the economic survey of Pakistan, these consumption patterns account for 79% of gross domestic product (Economic Survey of Pakistan; 2017–2018). Being a basic necessity, food captures a large share of consumers' consumption expenditure (Shah et al., 2018), and approximately 82% share of the household's consumption patterns are amalgamated into food consumption, having prominent influences on a nation's economic growth (Mudassar et al., 2012; Ullah, 2018). Henceforth, many prior researchers estimated the consumption patterns of individuals for different food items, such as meat (McAfee et al., 2010), dairy products (Akaichi and Revoredo-Giha, 2014), sweet berries (Zamora et al., 2021), grains (Ullah, 2018), honey (Hambaryan, 2021), and other food commodities (Vu, 2020). They observed that a change in the price and income has projecting effects on the individuals' food consumption patterns (Mudassar et al., 2012; Ullah, 2018; Hina et al., 2021).

Nevertheless, notably, people want to spend most of their money on nutritious food (Hayat et al., 2016), and for an improved living standard, people want to consume righteous quantities of various food items, referred to as “a balanced diet or a rich nutritious diet.” Meat, pulses, vegetables, and fruits are rich in nutrition (Udoh et al., 2013), and consumption of their righteous quantities can palliate nutritional deficiencies by providing a rich amount of calcium, iron, protein, and vitamin A (Binnie et al., 2014). However, people normally face dietary diversity while searching for a rich nutritious diet. Thus, the role of food in providing nutrients to an increasing population cannot be over stressed, as they highly depend on the income and budget shares of the households. For instance, the consumption pattern of some food items is more sensitive to the change in income and prices, whereas others are less sensitive. For example, when an upsurge in prices of a group of commodities occurs, households (particularly poorer ones) substitute it with the other group of commodities (Ullah and Jan, 2016), resultantly affecting their food consumption patterns. Researchers found that various traditional (income, relative prices, and own prices) and non-traditional (nutritional needs, food safety information, and diet) factors exist behind the dietary diversity and changing consumption patterns.

However, triangulating the discussion from above, we perceive that determining how people respond to the change in prices and income to maintain a viable consumption pattern is a challenge for researchers. To answer this, demand analysis is crucial. The reason is that normally, demand is deliberated as the willingness and ability of different individuals to consume various food items, such as meat, vegetables, pulses, and fruits. Considering the importance of demand analysis, we attempt to explore the factors affecting food demand patterns of households, specifically in Pakistan. The study seeks to address several other objectives, estimating own price and cross-price elasticities and expenditure and income elasticities to determine the influence of relative change in price, total expenditure, and income on the relative change in demanded quantities of the selected food products.

After reviewing the available literature on the Linear Approximate Almost Ideal Demand System (LA/AIDS) model, we make the following inferences. First, we observe that many researchers tested the theoretical applications of the LA/AIDS model using different data sets (e.g., time-series data, survey data, and primary data). Surprisingly, the researchers who worked on food demand concluded that people change their consumption patterns after a specific period of time because of the change in price or income. However, we found that this is not always the case and argued that a change in consumption patterns can occur at any time, even on a monthly, weekly, or daily basis. Second, we found that little work has been undertaken by the previous researchers to validate the LA∖AIDS model in the context of Pakistan. To the best of our knowledge, the question of how Pakistani households respond to the change in prices and income to maintain a viable food consumption pattern is still unanswered. Third, we found that vast literature has been found on different food consumption patterns. However, to the extent of the authors’ knowledge, none of the researchers estimated the food demand patterns by taking meat, vegetables, fruits, and pulses as different food baskets, specifically for the case of Pakistan. Therefore, we intend to extend the previous work by validating the LA/AIDS model for the case of the food market. The key difference between the present study and existing ones is that this study validates the LA/AIDS model in Pakistan by analyzing the food demand using meat, vegetables, fruits, and pulses as different food baskets for the first time by using the latest data of household integrated economic survey (HIES) from 2018 to 2019. Thus, we aim to estimate own price and cross price elasticities, and expenditure and income elasticities to show the influence of relative change in price, total expenditure, and income on the relative change in demanded quantities of the selected food products. The study further intends to answer how people substitute one commodity with another to maintain a viable food consumption pattern. The contribution of the present study not only enhances the available literature but also deepens the understanding of the practical applications of the LA/AIDS model. This study also helps policymakers of Pakistan in establishing effective food policies.

## 2 Literature review

Deaton and Muellbauer (1980) suggested that LA/AIDS is among the extensively used flexible demand models that several researchers have widely studied. This section provides a comprehensive overview of the existing studies to obtain a deep understanding of this model to analyze consumer behavior. The literature documented several studies, but we reviewed some important ones.

### 2.1 Studies in international context

Mergos and Donatos (1989) conducted research under the theoretical lenses of the LA/AIDS model to investigate the demand for food in Greece. They collected annual time series data on food expenditure between 1950 and 1986. The results of their study showed that Greek consumers are more likely to increase their demand for beef and various livestock products during the period of high-income growth. Thus, they concluded a positive association between income and demand for the chosen products. Taljaard et al. (2003) analyzed the demand for chicken, mutton, pork, and beef for the case of South Africa during the period of 1970–2000. Under the applications of the LA/AIDS model, the study showed that demand for chicken is inelastic as it is a necessity good. The study further showed that mutton (beef) is a luxury (near luxury) good, and all these items have elastic demand.

Jabarin (2005) observed the meat consumption patterns of the Jordan households using micro survey data. The author applied a censored estimation approach to notice the consumption patterns of selected households. The study used a two-step approach to estimate the findings. First, the author used the probit model to analyze the inverse mill ratio. Then, he incorporated the estimated variables into the LA/AIDS model to explore the demand elasticities of meat. Prior to this, three assumptions of demand theory (i.e., homogeneity, symmetry, and adding up restrictions) were satisfied. The results of cross-price elasticities showed that mutton is substituted by beef and poultry. The results of expenditure elasticities indicated that fish and poultry are necessity food items, whereas beef and mutton are luxury food items. Overall results pointed out that the demand for poultry and mutton is more elastic, whereas it is less elastic for fish and beef.

Zamora et al. (2021) conducted a demand analysis, and the notable strength of their work is that they had not only explored the demand for berries but also discovered the factors having a significant influence on the changing patterns of berries’ demand for Mexican households. They collected microdata from the national income expenditure survey 2018 for its formation of the LA/AIDS model. The findings of the study exhibited that the demand for berries in Mexico is more elastic (shown by Marshallian elasticity) and a luxury good (shown by expenditure elasticity). In addition, the study found that sweet fruits are a substitute product for berries, whereas melons and semi-acidic fruits are complementary to berries (shown by Marshallian cross-price elasticity). The study concluded that changes in prices of berries and income patterns of the people are the crucial factors with a significant influence on the changing demand patterns of berries for Mexican households.

Khoiriyah et al. (2020) examined how the change in price, income, and household size affect the demand patterns of eggs, chicken, milk-power, beef, and fish for the case of Indonesian households using the LA/AIDS model. In this regard, they collected data from a socio-economic survey of Indonesia in 2016. The results exhibited a positive income elasticity, whereas a negative price elasticity for all the selected products. The results further demonstrated that the demand for chicken, beef, and fish is more elastic compared with milk powder and eggs.

Basarir (2013) conducted a study on meat consumption in UAE by categorizing the meat into six different classifications (i.e., goat, chicken, camel, fish, beef, and lamb). The author was interested in determining meat demand patterns of different UAE households. In this regard, the data from 600 randomly selected households were gathered through a structured interview. The study applied the outwardly unrelated regression model under the theoretical support of the LA/AIDS model to scrutinize the empirical results. The outcomes of the study reflected that UAE green card holders demand more beef, whereas civilized households prefer lamb. The study found that an increase in household size is positively correlated with the demand for camel meat. By contrast, the increase in income is positively correlated with the demand for goat meat but negatively correlated with the demand for beef. The results of Hicksian and Marshallian elasticity pointed out that all the selected meat products are less sensitive to price changes. The outcomes of cross price elasticity depicted that camel and chicken substitute for each other, whereas beef and goat, camel and fish, and goat and chicken are complementary. Expenditure elasticity showed that goat, lamb, and camel are luxury, whereas fish and chicken are necessity products.

Adejobi et al. (2014) aimed to explore the demand patterns for poultry products. They acquired data from the respondents through a structured questionnaire using a two-way sampling approach. After empirically estimating the collected data under the theoretical support of the LA/AIDS model, the study found a negative relationship between price and demand for poultry products, whereas a positive relationship between income and demand for poultry products. The study further found that the demand patterns are more sensitive to the change in prices and income for the poorer people.

### 2.2 Studies in the context of Pakistan

Hayat et al. (2016) conducted a study in Pakistan and observed food demand patterns of Pakistani households. In this regard, they collected data from the Pakistan panel household survey in 2010 and used the LA/AIDS model to evaluate demand elasticities. The results of the expenditure elasticity depicted that sugar, grains, ghee, vegetables, and pulses are necessities, whereas meat and milk are luxury goods. The results of uncompensated cross-price elasticities reveal that ghee and meat, vegetable and pulses, and milk and sugar are complementary goods. Moreover, meat, vegetables, and pulses are substitute goods for one another. They further showed that an increase in income (household size) is positively (negatively) correlated with the demand for meat and milk products.

Malik et al. (2015) took the support from the quadratic aids (QA/AIDS) model and explored food consumption behavior among Pakistani households. For this purpose, they collected data from HIES from 2010 to 2011 and found significant expenditure elasticities among the selected food items. The study further revealed a positive connection between income and the demand for fruits and milk products. The study found that wheat and rice are necessary items and have less elastic demand.

Sher et al. (2021) also performed demand analysis on the food commodities of Pakistan by collecting the data from “Pakistan Social and Living Standard Measurement Survey, 2007–2008.” The study analyzed income elasticities and household size to examine food consumption patterns. The findings of the study exhibited that the income elasticities are positive. Akram (2020) proposed a conceptually identical work, utilizing a different but similar method. The author also conducted a demand analysis on different food commodities of Pakistan by estimating the QA/AIDS model. The study collected data from a “household integrated income and consumption survey” to estimate the empirical results. The findings indicated that fresh juices, dry fruits, soda water, bakery products, mutton, seafood, and beef are luxury products. Milk, pulses, tea, vegetables, oil, rice, and yogurt are necessities. The estimations of price elasticities depicted that the demand for beef, soda water, seafood, eggs, and mutton is more elastic compared with other commodities.

Hina et al. (2021) calculated the demand for milk in Karachi, the largest city in Pakistan. The study extracted the data from the HIES survey from 2005 to 2006. They incorporated the effect of socio-economic variables into the model. The results showed that the demand for milk is more price sensitive as it is a luxury good for Karachi residents. The study further showed a positive connection between education and level of income and milk. The study reports that people tend to purchase more milk as their income level and level of education arise. Haq et al. (2011) analyzed the demand for food items in the province of Punjab. After applying the LA/AIDS model by utilizing the data of the HIES survey, the study found a significant and positive influence of change in price and income on demand patterns of the chosen commodities.

### 2.3 Development of hypotheses

After reviewing the above literature, we found that many researchers reached the conclusion that changes in income and price of a product and its substitutes have a significant influence on product demand. The possible justification behind this notion is that when a change occurs in the price of a product, consumers tend to reduce the quantity demanded (QD) as per the law of demand (Al Rawashdeh, 2022). Another justification is that the increase in prices reduces the purchasing power of the consumers (Zhang et al., 2020). Hence, they are more likely to consume a lesser amount of that product (Erutku, 2005). Similarly, the effect of change in demand also occurs if consumers face change in the substitutes of a product. For instance, a decrease (increase) in the price of a substitutable commodity attracts the attention of consumers to consume more (less) of that product (Levis and Papageorgiou, 2009), as it will increase (decrease) their purchasing power (Zhang et al., 2020). The effect of change in income on the change in demand is also justified easily. It is argued that a change in income leads to a substantial amount of change in demand. The reason is that if income increases, consumers become capable of consuming those commodities that were beyond their former budget (Kemp, 1998). Thus, we hypothesize the following:

H1 A change in income has a positive influence on the demand patterns of a particular commodity.

H2 A change in price has a negative influence on the demand patterns of a particular commodity.

H3 A change in price of the substitute product has a positive influence on the demand patterns of a particular commodity.

## 3 Methodology

### 3.1 Demand model

The present study applies the LA/AIDS suggested by Deaton and Muellbauer (1980) to estimate the demand for selected food products (e.g., demand for meat, vegetables, pulses, and fruits).

LA/AIDS is among the extensively used flexible demand model and is widely studied by several researchers (Taljaard et al., 2003; Adejobi et al., 2014; Green and Alston, 1990; Alnafissa, and Alderiny, 2020). This model is obtained by the cost function, which shows the minimal cost required to achieve a given level of utility (U) at a price level (P). The cost function is shown in Eq. (1).

(1) $$\ln\;C\;\left(U,\;P\right)=\;\beta_{0}+\overset{n}{\underset{1}{\sum }}\beta_{i}lnP_{i}+\;\frac{1}{2}\overset{n}{\underset{i}{\sum }}\overset{n}{\underset{i}{\sum }}\delta_{ij}lnP_{i}lnP_{j}+u\alpha_{0}\underset{i}{\prod }{p_{i}}^{\alpha_{i}},$$

where *i* = 1,2,3,4.

In Eq. (1), ln C (U,P) is the cost function; $\beta_{0},\beta_{i},\;\alpha_{0},\;\alpha_{i},\;and\;\delta_{ij}$ are the constant parameters; i and j are the catalogs that symbolize different food categories. However, the adjusted version of the AIDS model can be derived from Eq. (1) by taking its first derivative with respect to price (e.g., $Q_{i}=\frac{\partial C\;(u,\;p_{i)}}{\partial p_{i}}$), or in other words, by applying shepherd's lemma (see Eq. (2), where the share of expenditure of i^th^ food category is a function of prices and the relative food expenditures).

(2) $$w_{i}=\beta_{i}+\sum \delta_{ij}lnp_{j}+\alpha_{i}\text{ ​ln}\left(\frac{X}{P}\right),$$

where.

• is the share of expenditure on *i*^*th*^ commodity, calculated as $\frac{p_{i}q_{i}}{\sum p_{i}q_{i}}$.
• *p*_*j*_ is the price of *j*^*th*^ good.
• X is the total expenditures on the selected food commodities.

P signifies the stones price index, which is specified as: $\ln\;P=\sum w_{j}lnp_{j}\beta_{i},\;\alpha_{i},\;and\;\delta_{ij}$ are predictable parameters: where; β_i_ is intercept, α_i_ signifies the effect of “real income” relative to a change in expenditure shares of a product, δ_ij_ deals with the effect of a “price change” of j^th^ commodity, relative to the expenditure share of i^th^ commodity.

### 3.2 Advantage of the demand model

The demand model is among the broadly utilized flexible model that has been extensively studied by numerous researchers (e.g., Alnafissa et al., 2020; Adejobi et al., 2014). The AIDS model, developed by Deaton and Muellbauer (1980), provides an alternate technique for estimating the demand. The benefits of the AIDS model include the following: (a) it offers “arbitrary first-order approximation” to the demand system, (b) it combines entirely on the consumers, (c) it perfectly satisfies the choice axiom, (d) it is simply estimated in the linear form, (e) it is utilized in testing the symmetry and homogeneity, and (f) it has a functional form that is consistent with the prior household data. In addition, being theoretically finer like other demand models, several implications of the AIDS model have twisted encouraging outputs. Considering the above-mentioned reasons, the AIDS model system has attracted the attention of recent studies (Megros and Donatos, 1989; Fulponi, 1989, among others).

### 3.3 Demand model restrictions

The LA/AIDS model is comprised of some restrictions that are mandatory for the theoretical consistency of Eq. (2). However, these restrictions are inevitably satisfied by the AIDS model (Moschini, 1998). These restrictions are stated as follows:

#### 3.3.1 Adding up restriction

The “adding up” restriction is presented in Eq. (3), which reports that the allocated share of expenditures for all commodities must be equal to unity (e.g., $\overset{n}{\underset{i=1}{\sum }}w_{i}=1$).

(3) $$\underset{i}{\sum }\beta_{i}=1;\;\underset{i}{\sum }\delta_{ij}=0;\;\underset{i}{\sum }\alpha_{i}=1.$$

#### 3.3.2 Homogeneity

The restriction of “homogeneity” is depicted in Eq. (4), showing that doubling the prices and incomes brings no change in the QD.

(4) $$\underset{i}{\sum }\delta_{ij}=0.$$

#### 3.3.3 Symmetry

The restriction of “symmetry” is specified in Eq. (5), which compacts with the substitution effect among the commodities. This condition shows the constancy in the choices of consumers.

(5) $$\delta_{ij}=\;\delta_{ji}\forall_{i}\;and\;i\neq j.$$

### 3.4 Price and expenditure elasticities

After satisfying the aforementioned restrictions, the next step is to calculate expenditure elasticities. The notation to calculate the expenditure elasticities is presented in Eq. (6).

(6) $$\eta_{i}^{E}=\;\frac{\alpha_{i}}{w_{i}}+1.$$

However, this study follows Asche and Wessells (1997) to calculate uncompensated (Marshallian) and compensated elasticities (Hicksian).

#### 3.4.1 Uncompensated price elasticities

Uncompensated price elasticity (e_ij_), generally known as Marshallian price elasticity, incorporates income and substitution effect, which is calculated using Eq. (7).

(7) $$\left(e_{ij}\right)=\;-r_{ij}+\frac{\delta_{ij}}{w_{i}},$$

$$\text{where}\;r_{ij}=\;\left\{\begin{array}{l} 1\;if\;i=j\;\\ 0\;if\;i\;\neq j \end{array}\right\}$$

#### 3.4.2 Compensated price elasticities

Compensated price elasticity (ce_ij_), generally known as Hicksian price elasticity, incorporates only the substitution effect, which is calculated using Eq. (8).

(8) $$ce_{ij}=\;\frac{\delta_{ij}-\alpha_{i}w_{j}}{w_{i}}-\;r_{ij}.$$

Thus, the respective own price, cross-price, and expenditure elasticities are the function of the estimated coefficients of the demand system and the expenditure shares.

### 3.5 Data and sources

Data collection is a crucial component of research, and acquiring data from reliable sources is crucial to obtaining desirable, effective, and efficient results. Therefore, this study acquired data from HIES of Pakistan in 2018–2019. The dataset presented in the study is cross-sectional in nature. The study has extracted data on quantity consumed and budget shares of 13713 households on selected food groups (meat, vegetables, fruits, and pulses) to estimate their demand.

## 4 Results and discussion

### 4.1 Descriptive statistics

Table 1 shows the results of descriptive statistics about the prices and budget shares of selected food groups. Table 1 also reports the value of mean and standard deviation (SD) along with the coefficient of variation (CV) for the selected food items. The output depicts that the values of SD of prices and budget shares do not lie far away from their mean value, implying that the problem of normality does not exist in the data. After the rigorous examination of the mean values of budget shares of different commodity groups, households spend a major proportion of their budget on the consumption of vegetables and the least proportion of their budget on the consumption of meat. The outcomes of CV reveal that fruits have the highest variations in the aggregate prices, whereas pulses have the least variations in the aggregate prices.

**Table 1:** Descriptive statistics.

Food categories	Summary Statistics	Prices	Budget shares
Meat	Mean	314.8403	12.7634
	SD	299.6904	10.6533
	CV	95.1880	83.4675
Vegetables	Mean	44.7464	33.7745
	SD	37.8585	29.7364
	CV	84.6067	88.0439
Fruits	Mean	184.7340	25.2293
	SD	98.8327	24.8744
	CV	95.3577	98.5933
Pulses	Mean	99.4483	19.8832
	SD	97.0117	16.8764
	CV	97.5494	84.8765

Note: CV: coefficient of variation, SD: standard deviation.

### 4.2 LA/AIDS model estimates

Table 2 reports the estimated parameters of the LA/AIDS model. These parameters are obtained by imposing the restrictions of homogeneity, additivity, and symmetry for the theoretical consistency of the model. The results show that all the parameters are statistically significant at the 1% level. Hence, we conclude with 99% confidence that the demand for a particular product is significantly influenced by the change in own price, cross prices, and real income. Thus, all the hypotheses are accepted.

**Table 2:** Parameters estimates of LA/AIDS model.

Commodity groups	Coefficients	Probability values
**Meat**		
Sigma-lnP_1_	-0.0435	0.000∗∗
Sigma-lnP_2_	0.0403	0.000∗∗
Sigma-lnP_3_	-0.0177	0.000∗∗
Sigma-lnP_4_	0.0209	0.000∗∗
alpha-lnX	0.8119	0.000∗∗
Beta-constant	0.7463	0.000∗∗
**Vegetables**		
Sigma-lnP_1_	-0.0265	0.000∗∗
Sigma-lnP_2_	0.2691	0.000∗∗
Sigma-lnP_3_	-0.1985	0.000∗∗
Sigma-lnP_4_	0.9460	0.000∗∗
alpha-lnX	0.3494	0.000∗∗
Beta-constant	0.0457	0.000∗∗
**Fruits**		
Sigma-lnP_1_	-0.2134	0.000∗∗
Sigma-lnP_2_	-0.3805	0.000∗∗
Sigma-lnP_3_	0.1586	0.000∗∗
Sigma-lnP_4_	0.9159	0.000∗∗
alpha-lnX	0.1642	0.000∗∗
Beta-constant	0.0035	0.000∗∗
**Pulses**		
Sigma-lnP_1_	0.0113	0.000∗∗
Sigma-lnP_2_	-0.0235	0.000∗∗
Sigma-lnP_3_	-0.0142	0.084∗
Sigma-lnP_4_	-0.2324	0.000∗∗
alpha-lnX	-0.8935	0.000∗∗
Beta-constant	0.9991	0.000∗∗

Note: ∗ and ∗∗ indicate level of significance at 10% and 1%, respectively.

Table 3 shows the mean square error of regression parameters of the LA/AIDS model, adjusted R^2^, and F-statistics, along with the probability values. From the results, the estimated results are accurate as the values of MSE for all the estimated regressions (i.e., meat, vegetables, fruits, and pulses) are low. Nonetheless, fruit items have the comparatively lowest value of MSE (0.0165 for fruits), whereas meat products have the comparatively highest value of MSE (0.0908 for meat). The results further showed that the explanatory power of the estimated equations ranges from 0.1931 to 0.5113 (shown by the value of R^2^). The value of R^2^ is low because we use cross-sectional data with a large number of observations. Significant F-statistics show that the overall models are a good-fit.

**Table 3:** Goodness of fit statistics.

Equation	MSE	R-square	F-statistics	P-value
Meat	0.0908	0.1931	656.1700	0.000∗
Vegetables	0.0531	0.2094	726.0600	0.000∗
Fruits	0.0165	0.5113	2868.370	0.000∗
Pulses	0.0276	0.3680	1596.0100	0.000∗

Note: a: significant at 1%.

### 4.3 Own price, cross price, and expenditure elasticities

We estimate expenditure elasticities, Marshallian, and Hicksian price elasticities. Panels A, B, and C of Table 4 show the results.

**Table 4:** Expenditure and price elasticities.

	Meat	Vegetables	Fruits	Pulses
Panel A: Expenditure elasticities				
$\eta_{i}^{E}$**=**$\frac{\alpha_{i}}{w_{i}}+1$	1.4642	0.4574	1.1955	0.1065
Panel B: Uncompensated own & cross price elasticities (Marshallian elasticities)				
	Meat	Vegetables	Fruits	Pulses
**Meat**	**-1.402∗∗∗**	+0.672∗∗	+0.163∗∗∗	-0.556∗∗
**Vegetables**	+0.778∗∗	**-0.616∗∗∗**	+0.345∗∗	-0.588∗∗∗
**Fruits**	0.381	0.387	**-1.641∗∗**	-0.510
**Pulses**	-0.453∗∗	-0.684∗∗∗	-0.179	**-0.455∗∗**
Panel C: Compensated own and cross price elasticities (Hicksian elasticities)				
**Food items**	Meat	Vegetables	Fruits	Pulses
**Meat**	**-1.1245∗∗**	+0.293∗	-0.113∗∗∗	+0.358∗∗∗
**Vegetables**	+0.293∗∗	**-0.453∗∗∗**	+0.139∗∗	-0.496∗∗∗
**Fruits**	+0.332∗	+0.012∗	**-1.213∗∗∗**	0.355
**Pulses**	-0.397∗∗	+0.676∗∗	+0.184	**-0.438∗∗∗**

Where: ∗, ∗∗ and ∗∗∗ donates the level of significance at 10%, 5% and 1% respectively.

#### 4.3.1 Expenditure elasticities $\left(\eta_{i}^{E}\right)$

Expenditure elasticities are synonymously used with income elasticities. Panel A of Table 4 presents the results of expenditure elasticities. Table 4 shows that the estimated $\left(\eta_{i}^{E}\right)$ of vegetables and pulses are less than 1 ($\eta_{i}^{E}<1)$, which implies that pulses and vegetables are “necessities.” However, the estimated $\left(\eta_{i}^{E}\right)$ of meat and fruits are greater than 1 $\left(\eta_{i}^{E}>1\right)$, which indicates that these food products are “luxuries.” The results state that the meat products and fruit items are more responsive to the change in real income compared with vegetables and pulses as they are more income elastic. People tend to increase the consumption of meat and fruits (luxury products) when there is a real increase in their income (Kemp, 1998). The reason is that most households in Pakistan have less purchasing power, and the largest share of their income goes to the fulfillment of their basic needs. Thus, they contribute every minute share of their income to luxury products (Besley, 1989). Households consider luxuries as “conspicuous consumption,” which refers to the purchase of goods exclusively to show off one's wealth. Hence, meat and fruits, being luxury products, capture very minute share of the budget from their income. However, when households face a real increase in their income, they are more likely to incline toward the consumption of conspicuous products (i.e., meat and fruits). The reason is that the increase in real income resultantly increases the purchasing power of the households (Sa'diyah et al., 2019). Hence, luxury goods are more sensitive to the change in income. However, the demand for vegetables and pulses is not much sensitive to the change in income as they are inelastic. The reason behind this is that vegetables and fruits are essential substances for households. As necessities, households cannot reduce the demand of necessity products even if they face a decline in their real income (Haque, 2006). People must have to purchase these commodities (vegetables and pulses) to meet their basic requirements, even if they have zero income. Put it differently, when people face an increase in their real income, they are not concerned about increasing the QD of their basic necessities as they are already receiving enough amount of satisfaction with previously attained quantities. Instead, now they are fascinated to increase the QD of those commodities, which were out of their previous budget (i.e., luxury products). Thus, necessity items are less sensitive to the change in income. The findings of the present study are consistent with those of several previous studies (Zamora et al., 2021; Hayat et al., 2016; Khoiriyah et al., 2020; Farooq et al., 1999; Weerahewa et al., 2013; Obisesan, 2021), who also concluded meat and fruit as a luxury, whereas vegetable and pulses as necessity commodities for households, specifically for developing economies.

#### 4.3.2 Marshallian elasticities $\left(e_{ij}\right)$

We estimate compensated (Marshallian) and uncompensated (Hicksian) own and cross-price elasticities. Panels B and C of Table 4 show the results. Hayat et al. (2016) argued that Marshallian elasticities provide a more accurate image of substitutes and complements compared with Hicksian elasticity. Therefore, we have only discussed own price and cross-price effects of Marshallian elasticity in detail.

##### 4.3.2.1 Own price elasticities (e_i_)

Panel B of Table 4 shows own price Marshallian elasticities (see bold diagonal values). According to the economic theory, own price elasticity of a commodity (e_i_) is likely to have a “negative” sign, which shows an inverse relationship between the price and QD. The magnitude of e_i_ tells us about the nature of the commodity (i.e., elastic or inelastic). A product is said to be as elastic if e_i_ > 1, whereas a product is said to be inelastic if e_i_ < 1. Table 4 shows that the own price elasticities for meat, vegetables, fruits, and pulses are −1.402, −0.616, −1.641, and −0.4554, respectively. According to the results, vegetables and pulses are “inelastic goods” as their own price elasticity is less than 1 (e_i_ < 1). Then, meat and fruits are “elastic goods” as their own price elasticity is greater than 1 (e_i_ > 1). The results imply that if there is a 1% increase in the prices of the selected food commodities, households will tend to reduce the QD of meat by 1.402%, fruits by 1.641%, vegetables by 0.616%, and pulses by 0.455%. The findings suggest that households are comparatively more sensitive to changes in the price of meat and fruits compared with vegetables and pulses as the percentage change in the QD of meat and fruits is greater than that in their prices. However, households are comparatively less sensitive to changes in the price of vegetables and pulses as the percentage change in the QD of these commodities is less than that in their prices. Many other researchers also concluded meat and fruits as elastic, whereas vegetables and pulses as inelastic goods (Mudassar et al., 2012; Zamora et al., 2021; Hayat et al., 2016; Han and Wahl, 1998).

##### 4.3.2.2 Cross price elasticities (e_ij_)

Cross price elasticity (e_ij_) measures the change in the QD of one product (e.g., product i) caused by the change in the price of another product (e.g., product j). It represents the relationship between two or more products and tells whether the products are substitutes or complements. A positive sign with e_ij_ indicates substitutability, whereas a negative sign indicates the complementary relationships among two or more items. Panel B of Table 4 shows the cross-price Marshallian elasticities. The results show that vegetables and meat and vegetables and fruits are substitutes for each other (see the positive sign with the elasticity coefficients). The possible justification behind this substitutability is that the aforementioned substitutable commodities provide an almost equivalent level of nutritional requirements (Kumar et al., 2011). The present study offers several arguments to justify the meat–vegetable substitutability. For instance, people substitute meat with vegetables and vice versa when they face a rise in the prices of one commodity. Evidence shows that vegetables and meat contain a sufficient amount of calories, which are mandatory to fulfill a minimum threshold of daily nutritional requirements. Therefore, when people face a rise in the prices of meat (vegetables), they substitute meat (vegetable) with vegetables (meat). Some people also substitute meat for vegetables because of the negative influence of high meat consumption on human health. A higher intake of meat increases the level of cholesterol and saturated fat, which is dangerous for human health. Hence, the people who are very health conscious substitute their consumption of meat with vegetables. An animal suffering is another reason for the substitutability between meat and vegetables (Gabriel et al., 2003). People often snoop about infectious or diseased chickens. Hence, they become conscious about the consumption of chicken and prefer the consumption of vegetables, which are hygienic and infection free comparatively. The substitutability between vegetables and meat is aligned with the findings of other researchers (Ullah and Jan, 2016; Ahmad et al., 2015; Tarrega et al., 2020).

The substitutability between vegetables and fruits is very surprising. Fruits and vegetables are decent substitutes for high energy density (Centers for Disease Control and Prevention, 2012), as both contain enough number of calories that are required to fulfill the minimum threshold level of a balanced diet. People can meet the minimum threshold level of daily nutritious intake by consuming either fruits or vegetables. Practically, when there is an increase in the prices of fruits, there is a decline in the prices of vegetables and vice versa. Hence, during the high prices of fruits, people prefer to substitute them with vegetables so that they may fulfill their basic dietary chucks. In addition to prices, some other reasons also exist why people substitute fruits with vegetables and vegetables with fruits. For instance, vegetables and fruits contain a large amount of vitamin C, which is a crucial ingredient of a balanced diet. People can easily substitute fruits with vegetables for the intake of this vitamin, for example, people might substitute orange, guava, or grapefruit with cauliflower, capsicums, and broccoli for the intake of vitamin C, specifically during the off seasons period of these fruits. Put it differently, suppose oranges contain a rich amount of vitamin C. In the summer season, when oranges are not easily available, people might substitute oranges with some vegetables to fulfill their nutritional requirement. Some behavioral factors also exist (e.g., taste and preferences of individuals), which persuade them to switch their consumption from vegetables (fruits) to fruits (vegetables). Some people are very diet conscious and want to consume hygienic food. As agriculture products, vegetables and fruits are very hygienic compared with other food (i.e., meat), as they are substitutable for the case of high price variations (or unavailability of one particular product).

The results further show that pulses and vegetables and pulses and meat are complementary products (see the negative sign with elasticity coefficients). Evidently, a difference exists between taking a diet and taking a healthy diet. If households consume pulses, then we are pretty sure that they are taking a diet but not the healthy one as pulses do not contain all the essential ingredients of a nutritious diet. One must consume pulses with meat or vegetables to fulfill the minimum threshold level of a nutritive diet. The findings are consistent with prior studies (Hayat et al., 2016; Andreyeva et al., 2010; Aziz et al., 2011).

### 4.4 Summary of results

Table 5 summarizes all the above findings, which clearly indicate that our results are in line with prior researchers. For instance, the results of expenditure elasticities show that vegetables (Hayat et al., 2016; Farooq et al., 1999; Obisesan, 2021) and pulses (Hayat et al., 2016; Farooq et al., 1999) are normal goods, whereas meat (Mudassar et al., 2012; Hayat et al., 2016; Sa'diyah et al., 2019) and fruits (Zamora et al., 2021; Weerahewa et al., 2013) are luxury goods. The results of uncompensated own price elasticities show that meat (Hayat et al., 2016) and fruits (Zamora et al., 2021; Han and Wahl, 1998) are elastic goods, whereas vegetables (Mudassar et al., 2012; Hayat et al., 2016) and pulses (Hayat et al., 2016) are inelastic goods. The outputs of uncompensated cross-price elasticities show that vegetables and meat (Ahmad et al., 2015; Tarrega et al., 2020) and vegetables and fruits (Centers for Disease Control and Prevention, 2012) are substituted goods, whereas pulses and vegetables (Hayat et al., 2016; Andreyeva et al., 2010) and pulses and meat (Hayat et al., 2016; Aziz et al., 2011) are complementary goods.

**Table 5:** Summary of results.

Food items(s)	Supporting Reference	Food item(s)	Supporting Reference
**Expenditure elasticities**			
**Normal goods**		**Luxury goods**	
Vegetables	(Hayat et al., 2016; Farooq et al., 1999; Obisesan, 2021)	Meat	(Mudassar et al., 2012; Hayat et al., 2016; Sa'diyah et al., 2019)
Pulses	(Hayat et al., 2016; Farooq et al., 1999)	Fruits	(Zamora et al., 2021; Weerahewa et al., 2013)
**Uncompensated Own price elasticities**			
**Elastic goods**		**Inelastic goods**	
Meat	(Hayat et al., 2016)	Vegetables	(Mudassar et al., 2012; Hayat et al., 2016)
Fruits	(Zamora et al., 2021; Han and Wahl, 1998)	Pulses	(Hayat et al., 2016)
**Uncompensated cross price elasticities**			
**Substituted goods**		**Complementary goods**	
Vegetable & Meat	(Ahmad et al., 2015; Tarrega et al., 2020)	Pulses & vegetables	(Hayat et al., 2016; Andreyeva et al., 2010)
Vegetable & Fruits	(Centers for Disease Control and Prevention, 2012)	Pulses & meat	(Hayat et al., 2016; Aziz et al., 2011)

## 5 Conclusions

We have conducted a consumer demand analysis on the food market of Pakistan, which has been a topic of pivotal importance for many years. Over the past few decades, this field has received sustained research activity of different academicians, even at present. Consumer demand analysis is an active and bourgeoning research area in developed and developing economies. We, therefore, attempt to contribute to the existing debate of consumer demand analysis by answering how Pakistani households respond to the change in prices and income to maintain viable food consumption patterns. Therefore, we have taken meat, vegetables, fruits, and pulses as different food baskets for the first time using the latest data of HIES in 2018–2019. We have also addressed other objectives. We estimated own price and cross price elasticities, and expenditure and income elasticities to show the impact of relative change in price, total expenditures, and income on the relative change in QD of the selected food products. We have applied LA/AIDS and acquired data from HIES of Pakistan in 2017–2018.

The outcomes of expenditure elasticity (uncompensated own price elasticity) reveal that vegetables and pulses are normal (inelastic goods), whereas meat and fruits are luxury items (elastic goods). This finding states that an increase in income (prices) will bring a substantial change in the QD of meat and fruits and a slight change in the QD of vegetables and pulses. The results of uncompensated cross-price elasticities reveal that vegetables and meat, and vegetables and fruits are substitutable commodities. This result implies that an increase in the price of vegetables will bring an increase in the QD of meat and fruits. From another aspect, pulses and vegetables, and pulses and meat are complementary goods, which means that we must have to purchase a combination of these products to attain a satisfactory amount of nutritional requirements. Thus, based on the findings, we suggest significant policies for the food policy makers of Pakistan.

### 5.1 Policy recommendations

First, we suggest that food policymakers must consider cross-price elasticities while imposing taxes on food commodities. For example, if the policymakers impose taxes on that product whose substitute is available in the market, then people will switch their consumption to its substitute because of the increase in prices. The study hence recommends that policymakers should not impose higher taxes on those food products whose substitutes are readily available in the market. Instead, they should impose taxes on those products that are complementary in nature so that households may not switch their consumption. Second, the policy makers must retain a well-known phrase “little drops make an ocean” in their minds while setting the prices of different food commodities. This result means that the policymakers should consider the welfare of individuals to promote the overall welfare of the economy. In this regard, we have offered the following recommendations. First, the policymakers have to set lower prices (i.e., those prices which are easily attainable for poor households) on the necessity products. This case will stretch the ease of access to the necessities to households, which will maximize their welfare that resultantly increases the overall welfare of the economy. Second, we recommend the policymakers impose higher taxes on the rich consumers (i.e., those consumers who have a higher income). Third, the study suggests introducing some schemes to provide income to the poorer households so that they can meet the nutritional food requirement.

Several developing economies also follow diverse food policies to ensure the sufficient availability of food to the residents to eradicate food shortage and malnutrition. For concern, China and India, being the two most populated economies, despite the prodigious economic growth, suffer from food insecurity (Yu et al., 2015). To meet the requirement of national food demand, both economies are dynamically promoting grain production through public stockholding, governmental procurement prices, and input subsidies (Yu et al., 2015). India mainly relies on its targeted public distribution system to make endowed grains available to the poor. Contrarily, China follows an all-inclusive income transfer program and non-food-based social safety net, which have been put into place to help the poor (Yu et al., 2015). Pakistan is following vertical urban development to evade the shortage of arable land that may lead to food shortages in the near future (Kousar et al., 2021). Moreover, Pakistan is an important country in one-belt-one-road, our findings will be fruitful for one-belt-one-road initiative countries like Pakistan and China, thus our results are also beneficial for China to enlarge two countries’ cooperation.

### 5.2 Limitations and future research directions

This study is a preeminent attempt to validate the LA/AIDS model in the context of the Pakistani food market, which provides satisfactory findings. However, we know that nothing is perfect. This study also has some limitations, which can be addressed by the upcoming researchers. First, this study has taken only four food baskets (i.e., meat, vegetables, pulses, and fruits) to provide the practical applications of the LA/AIDS model in the food market of Pakistan. However, future researchers can incorporate other food baskets to further enlighten the results of this study. Second, this study was conducted on the households of Pakistan. However, the food consumption pattern varies across the nations. Future researchers can conduct a replica of this study on the households of different developed and developing states. They can also conduct cross-nation comparisons to elucidate the behavior of global consumers. Third, the current study has tested the role of only traditional factors (income, relative prices, and own prices) on the changing demand patterns of consumers. Many non-traditional factors also exist (nutritional needs, food safety information, and diet), which may significantly influence the consumer's demand. Upcoming researchers can incorporate such factors to obtain more robust findings.

## Declarations

### Author contribution statement

Ghulam Mustafa: Conceived and designed the experiments; Performed the experiments; Contributed reagents, materials, analysis tools or data; Wrote the paper.

Weidong Huo: Analyzed and interpreted the data; Contributed reagents, materials, analysis tools or data; Wrote the paper.

Amber Pervaiz: Conceived and designed the experiments; Performed the experiments; Analyzed and interpreted the data; Contributed reagents, materials, analysis tools or data; Wrote the paper.

Muhammad Rizwan Ullah: Performed the experiments; Analyzed and interpreted the data; Contributed reagents, materials, analysis tools or data; Wrote the paper.

Muhammad Zulfiqar: Analyzed and interpreted the data; Contributed reagents, materials, analysis tools or data; Wrote the paper.

### Funding statement

This research was supported by 10.13039/501100018617Liaoning Revitalization Talents Program (XLYC2002116).

### Data availability statement

Data will be made available on request.

### Declaration of interest's statement

The authors declare no conflict of interest.

### Additional information

No additional information is available for this paper.
